# Predictors of childhood vaccination uptake and timeliness: a cross-sectional study in a diverse urban UK population

**DOI:** 10.3399/BJGP.2025.0319

**Published:** 2026-02-10

**Authors:** Karol Basta, Hiten Dodhia, James Crompton, David Whitney, Petrina Chu, Rachael Kilner, Ese Iyasere

**Affiliations:** 1 Public Health Department, London Borough of Lambeth, London, UK; 2 Department of Population Health Sciences, School of Life Course & Population Sciences, King's College London, London, UK; 3 NHS South East London Integrated Care System, Lambeth Civic Centre, London, UK; 4 Department of Biostatistics and Health Informatics, Institute of Psychiatry, Psychology, and Neuroscience, King’s College London, London, UK; 5 Herne Hill Road Medical Practice, London, UK

**Keywords:** child health, ethnicity, health inequities, immunisation programmes, primary health care, socioeconomic factors

## Abstract

**Background:**

Vaccination is a cornerstone of childhood public health. However, uptake has declined across the UK, with urban, deprived, and ethnically diverse communities facing unique barriers, exacerbated by the COVID-19 pandemic.

**Aim:**

To identify socioeconomic and clinical predictors of incomplete and untimely childhood vaccination between 2010 and 2023.

**Design and setting:**

This was a cross-sectional study using electronic health records from 40 general practices in Lambeth, an ethnically diverse London borough.

**Method:**

Routinely collected data for 37 912 children eligible for vaccines by age 5 years were analysed. Multivariable logistic regression assessed associations between predictors and (a) vaccination uptake and (b) timeliness, defined as receipt within 3 months of the scheduled age.

**Results:**

Of eligible children, 61.0% (*N* = 23 119/37 912) were fully vaccinated; of these, 60.8% (*N* = 14 062/23 119) were vaccinated on time. Lower uptake was associated with deprivation (most versus least deprived: adjusted odds ratio [AOR] 0.66, 95% confidence interval (CI) = 0.51 to 0.84), born outside the UK versus UK born (AOR 0.10, 95% CI = 0.08 to 0.12), and ethnicities other than White British (for example, Black Caribbean: AOR 0.30, 95% CI = 0.25 to 0.36). Children with clinical comorbidities had higher uptake (for example primary care-managed comorbidity: AOR 1.58, 95% CI = 1.46 to 1.71). Timeliness patterns differed: among those vaccinated, children born outside the UK and those from African, Caribbean, and Mixed ethnic backgrounds had higher odds of timely vaccination.

**Conclusion:**

Different predictors for uptake and timeliness highlight the need for strategies addressing both access and timely delivery. Local data should inform targeted, place-based interventions. Culturally competent services, integrated into routine primary care and codesigned with communities, are recommended.

## How this fits in

Childhood vaccination rates have declined in the UK, with inequalities in urban, deprived, and ethnically diverse populations. Previous studies have lacked individual-level clinical data or did not explore both uptake and timeliness. This study analysed 13 years of routinely collected primary care data for over 37 000 children in a diverse London borough to identify predictors of uptake and timeliness. Distinct sociodemographic and clinical factors were associated with incomplete and delayed vaccination, offering timely insights as responsibility for vaccination services shifts closer to local systems and place-based commissioning.

## Introduction

There is strong policy and clinical consensus on the benefits of childhood vaccination. However, over the past decade, UK childhood vaccination uptake has declined, with all vaccination rates falling below the World Health Organization’s 95% target in 2023–2024.^1^ A 2017 national Health Equity Audit (HEA) revealed avoidable inequalities in the programme, with vaccine-preventable diseases disproportionately affecting children living in deprivation.^2^ Since then, the COVID-19 pandemic has exacerbated existing challenges, increasing vaccine hesitancy in some communities and deepening mistrust in healthcare systems.^3^ Renewed efforts to ensure equitable vaccination are therefore integral for communicable disease control. This includes not only maximising overall uptake, but also ensuring timely delivery. Timely vaccination is particularly critical in early childhood when delays can leave children unprotected during periods of highest susceptibility to vaccine-preventable diseases.^4^

Urban, socioeconomically deprived, and ethnically diverse areas face distinct barriers to vaccination. Key contributing factors include a lack of trust in health services, low vaccine literacy compounded by language barriers, financial or logistical difficulties in accessing immunisation services, and negative perceptions regarding vaccine safety.^3^

Local HEAs are recommended as systematic tools to identify where and for whom the system is underperforming, enabling targeted, place-based action.^5^ Robust local intelligence will become increasingly important to support equitable vaccination delivery amid plans to transfer commissioning responsibilities from NHS England to local Integrated Care Boards (ICBs).^6^ Therefore, in a multiethnic UK urban setting, the aim of the study was to identify and quantify socioeconomic and clinical predictors of routine childhood vaccination uptake and timeliness over a 13-year period (2010–2023).

## Method

### Study design and population

Pseudonymised electronic health records (EHRs) from all 40 general practices in Lambeth, an ethnically diverse London borough, were analysed. Data were extracted from EMIS Web, the EHR system used by Lambeth practices, containing patient-level clinical and demographic information entered by GP staff.^7^ On 26 May 2023, data were extracted for 49 522 children born between 2010 and 2023 (aged 0–13 years) with an active GP registration

### Vaccination outcome measures

Uptake of vaccines included in the UK’s 2010 routine immunisation schedule for children <5 years of age were assessed.^8^ Vaccines introduced after 2010 (such as rotavirus, meningococcal group B [MenB], influenza) were excluded. In addition to individual vaccines, three combined outcomes were analysed based on routine schedule groupings and local qualitative insights suggesting predictors for measles, mumps, and rubella (MMR) may differ:

- all vaccines due <5 years: 6-in-1/5-in-1, pneumococcal conjugate vaccine (PCV), haemophilus influenzae type b (Hib)/meningococcal group C (MenC), MMR1, MMR2, 4-in-1;
- all vaccines due <5 years excluding MMR: 6-in-1/5-in-1, PCV, Hib/MenC, 4-in-1; and
- all vaccines due <2 years: 6-in-1/5-in-1, PCV, Hib/MenC, MMR1.

Uptake was assessed among only age-eligible children according to the routine schedule (Table 1).^8^ For example, for the complete uptake analysis of the 49 522 children extracted, 11 610 were excluded from this analysis as they were aged <3 years and 4 months at the time of extraction and were therefore ineligible to have received all routine childhood vaccinations. This left 37 912 eligible for this analysis. Changes in vaccine schedules, formulations, and combinations between 2010 and 2023 were accounted for.^8^ To assess data quality, extracted uptake estimates were compared with national COVER data^1^ and found to be consistent across all years.

**Table 1.** Vaccines included in the analysis

Vaccine name	Scheduled age as per UK childhood vaccination programme^8^
DTaP/IPV/Hib (5-in-1) or DTaP/IPV/Hib/HepB (6-in-1)	Three doses given at: 8, 12, and 16 weeks
PCV	If born before 1 January 2020: three doses given at 8 weeks, 16 weeks, and 12 months. If born after 1 January 2020: two doses at 12 weeks and 12 months
Hib/MenC	Twelve months
MMR1	Twelve months
MMR2	Standard schedule: 3 years and 4 months Accelerated schedule: 15 months from 2008 to 2017, 18 months from 2018
DTaP/IPV (4-in-1)	Three years and 4 months

a *In response to measles outbreaks in the late 2000s, seven boroughs in South East London, including Lambeth, adopted an accelerated MMR2 schedule. Starting in 2008, MMR2 was offered from 15 months or 18 months, depending on the borough. By 2012/2013, all seven boroughs were following the accelerated schedule. In Lambeth, the second dose was given at 15 months until 2018, when it was changed to 18 months*.^25,26^ DTaP = diphtheria, tetanus, pertussis vaccine. HepB = hepatitis B. Hib = Haemophilus influenzae type B. IPV = Inactivated Polio Vaccine. MenC = Meningococcal Group C. MMR = measles, mumps, rubella vaccine. PCV = pneumococcal conjugate vaccine.

Timeliness was defined as receiving the vaccine(s) within 3 months of the scheduled due age (Table 1). This threshold was agreed following consultation with local multidisciplinary and multiagency vaccination stakeholders. It was considered long enough not to be affected by common, short-term delays, such as minor illness or family travel. These delays are often outside the control of families or services. A 3-month window was therefore seen as a meaningful and actionable indicator of service performance and parental engagement. The analysis compared timely versus delayed vaccination among those vaccinated.

See Supplementary Table S1 for SNOMED codes used to define vaccination status.

### Covariates

Extracted sociodemographic variables included date of birth, sex, GP practice, year of registration, ethnicity, country of birth, interpreter need, and child protection status. Deprivation was measured using the 2019 Income Deprivation Affecting Children Index (IDACI) national quintiles.^9^ Detail on covariate definitions is provided in Supplementary Box S1.

Long-term comorbidities were extracted from diagnostic codes. To be considered relevant to prediction of vaccination status, diagnoses were only included if occurring before age 5 years. Comorbidities were grouped to explore if child engagement with primary or secondary care services had an impact on vaccination status (Box 1).

Box 1. **Grouping of comorbidities**

**Comorbidity group**

Childhood comorbidity usually treated in primary care as per NICE guidelines^27^
Neurodevelopmental comorbidity
Comorbidity or medication use that may be considered a contraindication to vaccination owing to immunodeficiency
Any other childhood comorbidity requiring referral to secondary care as per NICE guidelines^27^

**Specific comorbidities included**

Asthma or eczema
Cerebral palsy, Down’s syndrome, learning difficulty
Cancer, chemoradiotherapy, immune dysregulation
Chronic kidney disease, chronic obstructive pulmonary disease, diabetes, heart failure, HIV, liver disease, inflammatory bowel disease, sickle cell disease, congenital heart disease, epilepsy

NICE = National Institute for Health and Care Excellence.

### Statistical analysis

Multivariable logistic regression was used to examine associations between predictor variables and vaccination outcomes. No issues with sparse data or multicollinearity were observed, so a full model was used. Adjusted models included sex, age, IDACI, UK-born status, interpreter need, child protection status, ethnicity, and comorbidity group. Standard errors allowed for correlation within GP practices to relax the assumption of independent observations.

Of 37 912 children eligible for all routine vaccinations, 75 with missing postcode data (and therefore no IDACI value) were excluded from adjusted models. This is because postcode is a central data field in EHRs and its absence may indicate broader data-quality concerns. This left 37 837 for complete uptake analysis. For all other covariates, ‘missing’ categories were included as separate levels in regression models. This made it possible in the current study to retain these individuals and explore whether missingness was itself associated with vaccination outcomes. The extent of missing data across all variables is reported in Tables 2 and 3. For timeliness outcomes, only vaccinated children were included.

**Table 2.** Characteristics of those vaccinated with all vaccines due <5 years, out of the eligible population (*N* = 37 912)

Characteristic	% of total eligible sample represented by each characteristic	Vaccinated/ eligible, *n*
**Biological sex**		
Male	50.4	11 557/19 120
Female	48.4	11 378/18 350
Missing	1.2	184/442
**Year of birth**		
2010	4.2	987/1606
2011	10.3	2386/3904
2012	10.3	2494/3897
2013	10.6	2607/3999
2014	10.0	2433/3793
2015	10.1	2452/3836
2016	10.0	2337/3790
2017	9.6	2333/3655
2018	9.5	2210/3584
2019	9.1	2162/3460
2020	6.3	718/2388
**IDACI quintile**		
1 (most deprived)	38.4	8511/14 548
2	34.9	7846/13 213
3	16.3	3958/6177
4	8.3	2237/3146
5 (least deprived)	2.0	533/753
Missing	0.2	34/75
**Country of birth**		
UK	32.0	8385/12 116
Outside of UK	6.2	409/2338
Missing	61.9	14 325/23 458
**Interpreter need code**		
No	97.2	22 68/36 862
Yes	2.8	434/1050
**Child protection code**		
No	99.3	22 983/37 654
Yes	0.7	136/258
**Ethnicity subgroup**		
White British	21.6	6157/8192
Irish	0.4	108/167
Other White	13.9	3096/5268
African	12.0	2758/4550
Caribbean	6.1	1130/2315
Other Black	5.7	1114/2151
Indian	1.1	260/425
Pakistani	1.3	311/487
Bangladeshi	0.8	228/299
Chinese	0.8	196/285
Other Asian	2.8	706/1054
White and Black African	2.0	486/761
White and Black Caribbean	3.7	775/1410
White and Asian	1.4	399/549
Other Mixed	5.2	1116/1973
Arab	0.3	44/104
Any other ethnic group	4.1	773/1553
Missing	16.8	3462/6369
**Comorbidity usually managed in primary care**		
No	71.9	15 646/27 244
Yes	28.1	7473/10 668
**Neurodevelopmental comorbidity**		
No	99.6	23 022/37 748
Yes	0.4	97/164
**Comorbidity or medication causing immunodeficiency**		
No	99.6	23 015/37 749
Yes	0.4	104/163
**Comorbidity usually managed in secondary care**		
No	98.0	22 640/37 172
Yes	2.0	479/740
**Total**		23 119/37 912

IDACI = Income Deprivation Affecting Children Index.

**Table 3.** Characteristics of children who received all scheduled vaccinations on time by age 5, among those vaccinated (*N* = 23 119)

Characteristic	% of total eligible sample represented by each characteristic	Timely vaccinated/ vaccinated, *n*
**Biological sex**		
Male	50.0	7007/11 557
Female	49.2	6937/11 378
Missing	0.8	118/184
**Year of birth**		
2010	4.3	558/987
2011	10.3	1318/2386
2012	10.8	1298/2494
2013	11.3	1368/2607
2014	10.5	1337/2433
2015	10.6	1351/2452
2016	10.1	1404/2337
2017	10.1	1610/2333
2018	9.6	1337/2210
2019	9.4	1792/2162
2020	3.1	689/718
**IDACI quintile**		
1 (most deprived)	9.7	1384/2237
2	17.1	2542/3958
3	33.9	4839/7846
4	36.8	4941/8511
5 (least deprived)	2.3	333/533
Missing	0.1	23/34
**Country of birth**		
UK	36.3	4793/8385
Outside of UK	1.8	213/409
Missing	62.0	9056/14 325
**Interpreter need code**		
No	98.1	14 798/22 685
Yes	1.9	264/434
**Child protection code**		
No	99.4	13 978/22 983
Yes	0.6	84/136
**Ethnicity subgroup**		
White British	26.6	3991/6157
Irish	0.5	63/108
Other White	13.4	2023/3096
African	11.9	1566/2758
Caribbean	4.9	548/1130
Other Black	4.8	598/1114
Indian	1.1	185/260
Pakistani	1.3	196/311
Bangladeshi	1.0	139/228
Chinese	0.8	142/196
Other Asian	3.1	514/706
White and Black African	2.1	302/486
White and Black Caribbean	3.4	448/775
White and Asian	1.7	286/399
Other Mixed	4.8	659/1116
Arab	0.2	27/44
Any other ethnic group	3.3	461/773
Missing	15.0	1914/3462
**Comorbidity usually managed in primary care**		
No	67.7	9468/15 646
Yes	32.3	4594/7473
**Neurodevelopmental comorbidity**		
No	99.6	14 009/23 022
Yes	0.4	53/97
**Comorbidity or medication causing immunodeficiency**		
No	99.6	14 004/23 015
Yes	0.4	58/104
**Comorbidity usually managed in secondary care**		
No	98.0	13 785/22 640
Yes	2.0	277/479
**Total**		14 062/23 119

IDACI = Income Deprivation Affecting Children Index.

Results are presented as conditional adjusted odds ratios (AORs), reflecting associations while holding all other covariates at their reference categories. Unadjusted estimates are available in the Supplementary Tables S2–19. Analyses were conducted using Stata 18.

## Results

### Baseline characteristics

Of the 37 912 eligible, 61.0% (*n* = 23 119) were fully vaccinated (Table 2). Most lived in the two most deprived IDACI quintiles (73.2%, *N* = 27 761/37 912) and the sample was ethnically diverse: White British (21.6%, *n* = 8192), Other White (13.9%, *n* = 5268), African (12.0%, *n* = 4550), Caribbean (6.1%, *n* = 2315), and Other Black (5.7%, *n* = 2151). Primary care-managed comorbidities were present in 28.1% (*n* = 10 668) of children.

Of those fully vaccinated, 60.8% (*N* = 14 062/23 119) were vaccinated on time (Table 3).

### Socioeconomic and clinical predictors of vaccine uptake

Predictors of complete vaccination are shown in Figure 1. The odds of complete vaccination were 9% higher among female than male children (AOR 1.09, 95% CI = 1.04 to 1.14). Uptake decreased with increasing deprivation: those in the most deprived IDACI quintile were 34% less likely to be fully vaccinated than those in the least deprived (AOR 0.66, 95% CI = 0.51 to 0.84). Those born outside the UK were 90% less likely to be vaccinated compared with those who were UK born (AOR 0.10, 95% CI = 0.08 to 0.12). Children on the child protection register had reduced odds of vaccination (AOR 0.72, 95% CI = 0.55 to 0.93). Those with missing data on sex, ethnicity, or country of birth had lower uptake. Interpreter need was associated with lower uptake in unadjusted analysis (OR 0.45, 95% CI = 0.40 to 0.51, Supplementary Table S2). However, the adjusted OR was closer to the null and indicated that no clear association between interpreter need and odds of uptake remained after accounting for important confounders (AOR 0.85, 95% CI = 0.68 to 1.06).

**Figure 1.**
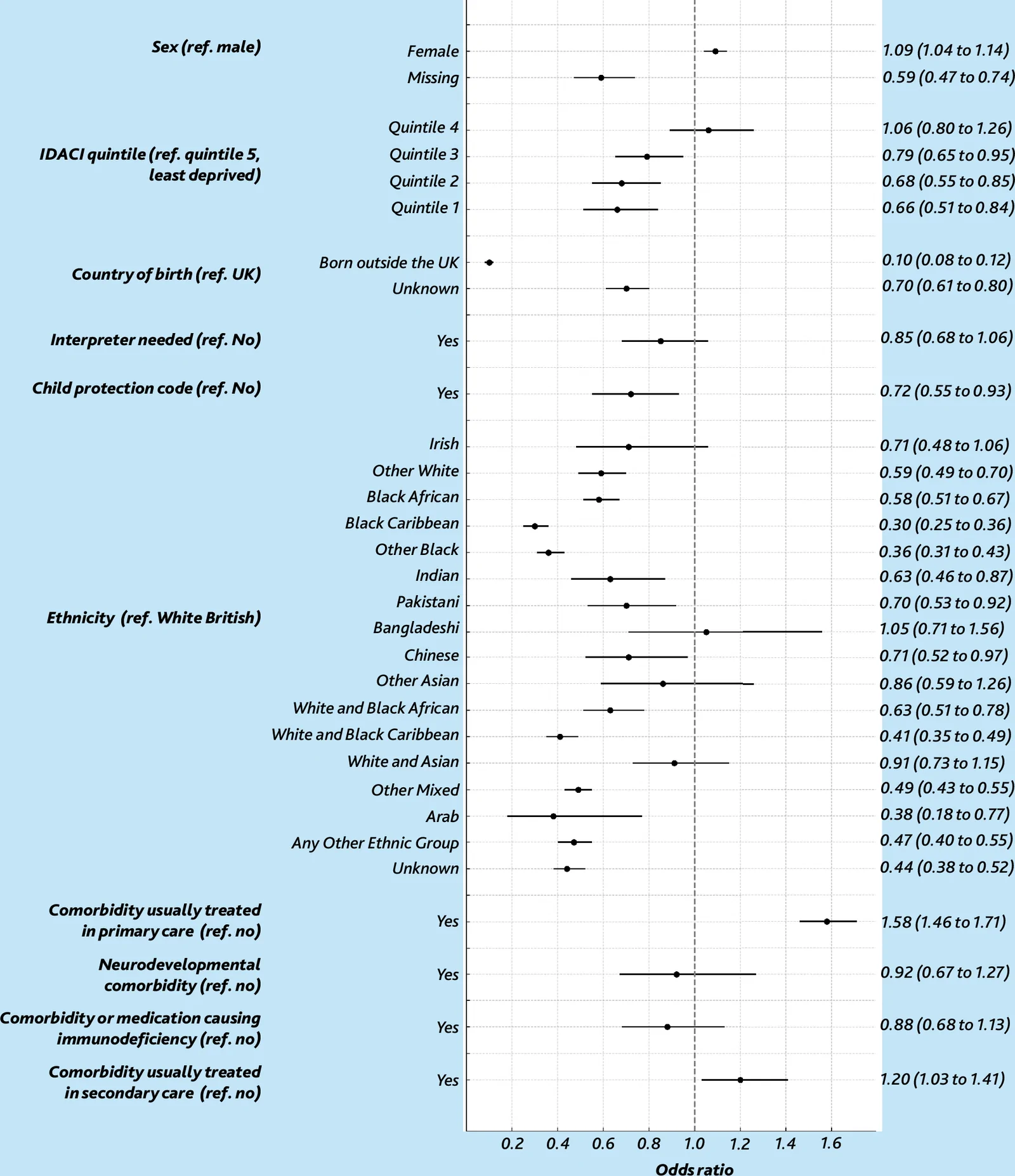
Sociodemographic and clinical predictors of overall vaccination uptake (*N* = 37 837). Adjusted odds ratios calculated using logistical regression model, presented with 95% confidence intervals. The adjusted odds ratios account for all predictors listed, as well as age at extract. Standard errors were adjusted for clustering by GP practice. Unadjusted odds ratios are available in Supplementary Table S2. IDACI = Income Deprivation Affecting Children Index. OR = odds ratio. ref. = reference category.

Compared with White British children, uptake was significantly lower across most ethnic minority groups, including: Other White, African, Caribbean, Other Black, Indian, Pakistani, Chinese, White and Black African, White and Black Caribbean, Other Mixed, Arab, and Any other ethnic group. No ethnic group had significantly higher uptake than the White British group.

Children with a primary care comorbidity had 58% higher odds of complete vaccination after adjustment (AOR 1.58, 95% CI = 1.46 to 1.71). Children with a secondary care comorbidity had 20% higher odds of complete vaccination compared with those without (AOR 1.20, 95% CI = 1.03 to 1.41). No significant associations were found for neurodevelopmental conditions or immunodeficiency.

Results for uptake predictors of other vaccine combinations and individual vaccines were consistent with overall uptake (Supplementary Tables S2–S10).

### Socioeconomic predictors and clinical predictors of vaccine timeliness

Predictors of overall vaccination timeliness are shown in Figure 2. Among vaccinated children, timely vaccination did not vary by sex, deprivation, interpreter need, or child protection status. Children born outside the UK had 44% higher odds of timely vaccination, compared with those born in the UK (AOR 1.44, 95% CI = 1.16 to 1.78). Compared with White British children, several ethnic groups had higher odds of timely vaccination, including African, Caribbean, Other Black, White and Black Caribbean, Other Mixed, Any other ethnic group, and Unknown (reflecting missing ethnicity data). Chinese and Mixed White and Asian children had lower odds of timely vaccination.

**Figure 2.**
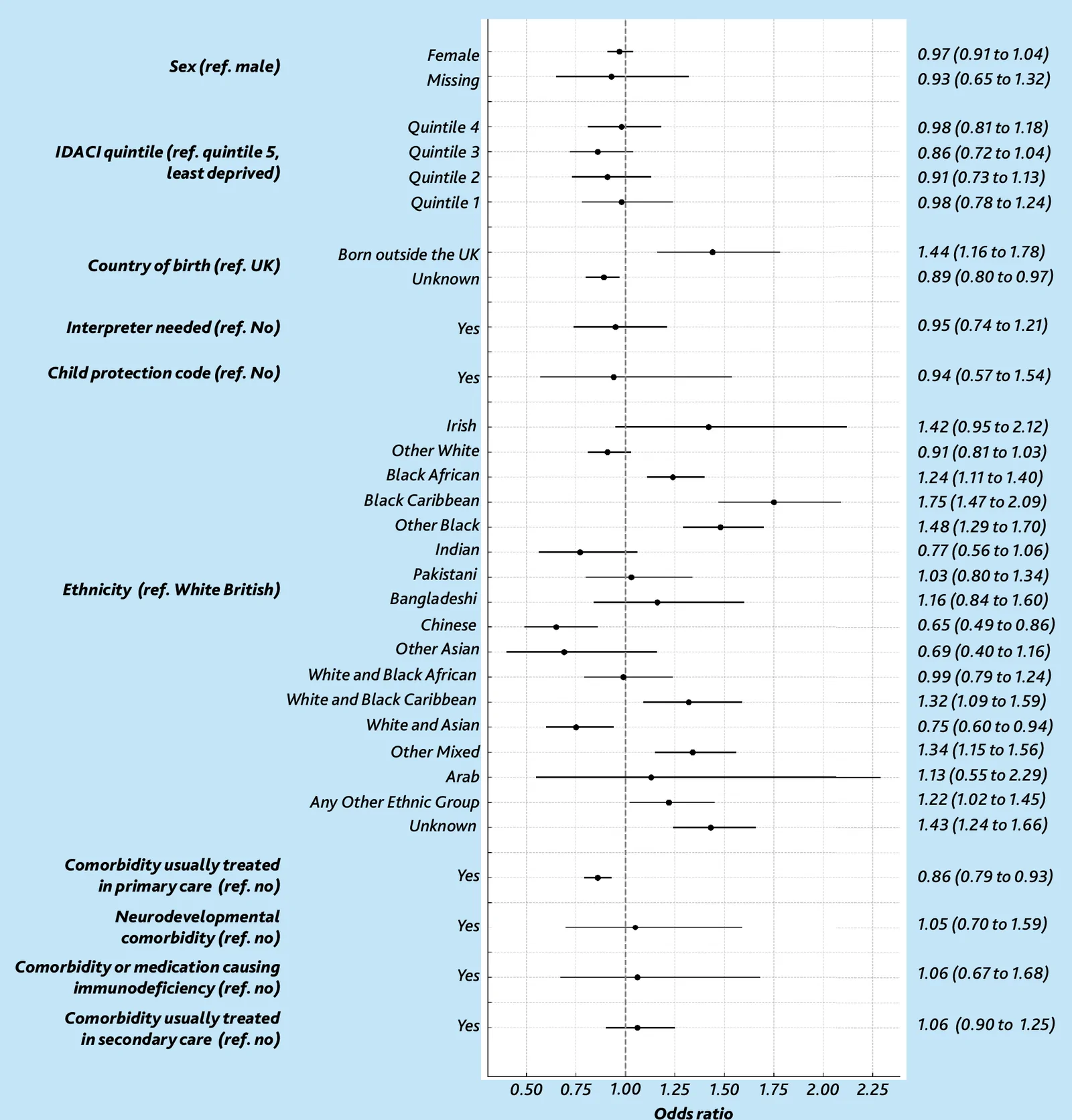
Sociodemographic and clinical predictors of overall vaccine timeliness (*N* = 23 085). Adjusted odds ratios calculated using logistical regression model. The adjusted odds ratios account for all predictors listed, as well as age at extract. Standard errors were adjusted for clustering by GP practice. Unadjusted odds ratios are available in Supplementary Table S11. IDACI = Income Deprivation Affecting Children Index. OR = odds ratio. ref. = reference category.

Additional results for individual vaccines and combinations are presented in Supplementary Tables S11–S19. Some differences in predictors emerged: female children had lower odds of timely vaccination for several individual vaccines (for example, 6-in-1, MMR1, PCV), although this pattern was not consistent across outcomes. Among MMR1 recipients, children in more deprived quintiles were more likely to be vaccinated on time. Interpreter need and child protection status were associated with improved timeliness for vaccines due by age 2 years but not by age 5 years. Ethnic predictors also differed by age, with higher odds of timeliness for those with Indian, Pakistani, and Mixed White and Black African backgrounds for vaccines due by age 2, but not by age 5 years.

After adjustment, children with primary care-managed comorbidities had 14% lower odds of timely vaccination compared with those without (AOR 0.86, 95% CI = 0.79 to 0.93) (Figure 2). No clear associations were found for other comorbidity types overall. However, children with secondary care-managed comorbidities had increased odds of timely pneumococcal vaccination (AOR 1.31, 95% CI = 1.05 to 1.63), and those with neurodevelopmental comorbidities had increased odds of timely 6-in-1/5-in-1 vaccination (AOR 1.46, 95% CI = 1.06 to 2.02) (Supplementary Tables S11–S19).

## Discussion

### Summary

In a multiethnic South London population, analysis of primary care data for 37 837 children revealed persistent inequalities in childhood vaccination uptake between 2010 and 2023. After adjustment, lower uptake was observed among children in deprived areas, born outside the UK, on the child protection register, and male children. Most minority ethnic groups had lower uptake than White British children. In contrast, children with comorbidities had higher odds of vaccination. These patterns were consistent across vaccine types.

Timeliness predictors differed. Children with primary care-managed comorbidities had higher overall uptake but lower timeliness. Conversely, those born outside the UK or from African, Caribbean, or Mixed backgrounds had lower uptake but, if vaccinated, were more likely to receive vaccines on time. Deprivation, interpreter need, and child protection status were not associated with timeliness by age 5 years.

### Strengths and limitations

This study used a large, population-based dataset spanning 13 years, enhancing robustness to policy and service changes over time. The sample’s diversity allowed detailed disaggregation of predictors, including ethnicity. This is a key strength, as broad ethnic categories may mask within-group variation and reduce the cultural and policy relevance of findings. Primary care EHRs provided a broad range of clinical and sociodemographic predictors, which have not previously been explored. Registration with general practices in the UK is free, widely encouraged, and not based on existing health conditions,^10^ minimising selection bias. As vaccine coding in EMIS web is linked to GP reimbursement through the Quality and Outcomes Framework, there is a strong incentive for complete and accurate recording.^11^ Uptake estimates were consistent with national COVER data, supporting the completeness and validity of the vaccination data used.

Less than 0.2% (*N* = 75/37 912) of the sample were excluded owing to missing postcode data. For other variables, including sex (1.2%, *N* = 442/37 912), ethnicity (16.8%, *N* = 6369/37 912) and country of birth (61.9%, *N* = 23 458/37 912), missing categories were included as separate levels in regression models. This made it possible in the current study to retain these individuals in the analysis and examine whether missingness itself was associated with vaccination outcomes. However, for country of birth, which has substantial missingness, estimates could be affected if missingness was not random and should be interpreted with caution.

The study was limited to children registered with a Lambeth GP at data extraction, excluding those who moved away or de-registered. Although prior research shows no systematic differences among frequent movers,^12^ this could underestimate missed vaccinations among more transient groups. Additionally, vaccines administered overseas or privately may not be updated in records. Despite adjusting for multiple covariates, unmeasured factors, such as parental education and household income, could have also influenced vaccination outcomes, introducing residual confounding. Some variables, including interpreter need and child protection status, may also be affected by underrecording or incomplete coding, and therefore may not capture all children with relevant needs.

The binary definition of timeliness (within 3 months of the scheduled age) does not differentiate between mild and substantial delays. Although children with severe contraindications were not excluded from the denominator, such cases are rare^13^ and unlikely to bias the results. The dataset did not allow exploration of reasons for non-uptake, and this study did not model pre- and post-COVID vaccination behaviour. Finally, although the study population is diverse, it is confined to a single urban borough, which may limit the generalisability of the findings to rural areas or other regions with different health systems.

### Comparison with existing literature

Previous UK studies have consistently found socioeconomic and ethnic inequalities^14–23^ in childhood vaccination, although few examined individual-level predictors of both uptake and timeliness in detail. A Scottish study reported declining uptake and timeliness with increasing area-level deprivation.^18^ Research in Wales using the Millennium Cohort Study found only modest differences in uptake by deprivation but more pronounced inequalities in timeliness for children born between 2013 and 2017.^19^ However, neither study adjusted for individual-level confounders or included clinical variables.

Lower uptake and timeliness were previously found among children from Black Caribbean, African, and Other Black backgrounds in London between 2001 and 2010.^20^ However, the study relied on administrative child health system data without individual-level clinical data, and it pre-dated the COVID-19 pandemic. Nationally, Clinical Practice Research Datalink (CPRD) data (2006–2021) showed lower uptake among children of Black Caribbean, African, and Other Black ethnicity, and higher uptake among children of Indian and Bangladeshi ethnicity, although timeliness was not assessed.^21^ A separate CPRD study found delayed vaccination among Black children between 2006 and 2014 but did not disaggregate by detailed ethnicity subgroupings.^22^

Evidence on clinical factors remains limited. The Millennium Cohort Study identified higher under-vaccination risk among children with intellectual disabilities.^24^ A Welsh cohort found associations between high birth weight, neonatal intensive care admission, and delayed vaccination,^23^ although this focused only on first-dose timeliness and did not account for wider clinical comorbidities or ongoing health needs.

The current study extends the literature by examining a wide range of sociodemographic and clinical predictors of both uptake and timeliness. Some findings contrast with previous studies; for example, it was observed that there was lower uptake among children of Indian and Pakistani ethnicity, which differs from findings in one national study^21^ and one local study.^16^ This divergence highlights the importance of locally led research and equity audits, particularly in diverse urban areas where national trends may obscure important place-based variation.

### Implications for research and practice

This study identified persistent inequalities by sex, deprivation, ethnicity, country of birth, and child protection status. Disaggregated ethnicity data revealed within-group variation that broader categories would obscure. Future research should use such data to inform targeted interventions. Vaccination pathways should prioritise cultural competence and co-design with underserved communities. Although some patterns reflected national trends, others diverged, highlighting the value of place-based approaches. As commissioning shifts to ICBs,^6^ access to granular local data will be essential for equitable delivery. In Lambeth, findings have already informed qualitative work and multiagency collaboration to design targeted interventions.

Although local action is essential, national policy must also account for structural disadvantage. Current payment models link funding to vaccination uptake,^11^ penalising deprived, diverse areas with lower rates. The current study’s findings suggest the greatest improvements in vaccination coverage could be achieved by increasing support for these areas, which require additional resources to overcome barriers to access.

Predictors of high overall uptake were often the opposite of those associated with timely vaccination. In groups with lower overall uptake, those who do get vaccinated may represent more engaged, health-literate, or well-supported families — who are also more likely to vaccinate on time. In contrast, higher-uptake groups may include a broader mix, including children vaccinated later. This suggests existing recall and catch-up systems may reinforce inequalities by better engaging some groups than others. Alternatively, families facing initial barriers may reliably follow the schedule once engaged. For non-UK-born children, higher timeliness rates may instead reflect selective registration of more engaged families rather than broader service reach. To further understand these patterns, future research could use survival methods to examine how quickly different groups receive vaccinations once eligible.

Higher uptake in children with comorbidities suggest healthcare contact as a key opportunity, but lower rates of timeliness highlights that access alone is not enough. Embedding vaccination into routine care and ensuring flexible delivery may improve timeliness. Given predictor consistency across vaccines, a unified approach to improving delivery and engagement should span the full childhood immunisation schedule.

## Data

Owing to data-sharing agreements with participating general practices, the pseudonymised datasets used in this study are not publicly available. Requests for access to the data can be directed to the corresponding author and will be considered on a case-by-case basis.
